# Long noncoding RNA LINC01638 contributes to laryngeal squamous cell cancer progression by modulating miR-523-5p/BATF3 axis

**DOI:** 10.18632/aging.202675

**Published:** 2021-03-10

**Authors:** Hang Zhang, Xudong Zhao, Mengmeng Wang, Wenyue Ji

**Affiliations:** 1 Department of Otolaryngology Head and Neck Surgery, Shengjing Hospital of China Medical University, Shenyang 110004, China; 2 The Sleep Medicine Center, Shengjing Hospital of China Medical University, Shenyang 110004, China

**Keywords:** laryngeal squamous cell carcinoma, LINC01638, miR-523-5p, BATF3

## Abstract

Long noncoding RNA (lncRNA) plays a critical role in tumorigenesis. How lncRNA regulates laryngeal squamous cell carcinoma (LSCC) progression remains poorly understood. In the present study, we found that LINC01638 was highly expressed in LSCC tissues. And LINC01638 expression was positively correlated with clinical stage and lymph node metastasis. Patients with LINC01638 high expression displayed a low survival rate. Results from CCK8, colony formation, and transwell assays showed that LINC01638 knockdown suppressed the proliferation, migration and invasion of LSCC cells *in vitro*. Animal experiments indicated that LINC01638 silencing attenuated tumor growth *in vivo*. In terms of mechanism, LINC01638 was found to sponge miR-523-5p and promote BATF3 expression. In summary, our results demonstrated that LINC01638/miR-523-5p/BATF3 axis plays a crucial function in initiating LSCC development and may be a potential target for tumor therapy.

## INTRODUCTION

Laryngeal squamous cell carcinoma (LSCC) is one of the most common cancers and belongs to the head and neck malignancies [1]. LSCC accounts for over 95% of laryngeal tumors. And a huge number of cancer-related deaths are induced by LSCC every year in the world [2]. Unfortunately, its incidence is still increasing. Although LSCC patients are treated by surgery, radiotherapy and chemotherapy, their prognosis remains very unsatisfactory [3]. The mechanism underlying LSCC recurrence and metastasis is largely unclear. At present, there is a very urgent need to identify novel therapeutic targets for LSCC patients.

Long noncoding RNA (lncRNA) is a subgroup of noncoding RNAs with over 200 nucleotides in length [4]. Although lacking protein-coding potential, lncRNAs play important roles in various biological processes, such as development, immune responses and cancer initiation [5, 6]. LncRNAs may regulate cell biology through chromatin remodeling [5]. Accumulating evidence shows that lncRNA participates in cancer initiation by forming a lncRNA-microRNA regulatory loop [6]. Aberrant expression of lncRNAs is usually observed in cancer and their dysregulation may affect cancer cell proliferation, migration, stemness and survival [7]. For example, lncRNA M26317 upregulation in gastric cancer is associated with poor prognosis and promotes cell growth and invasion [8]. LncRNA OTUD6B-AS1 is upregulated in clear cell renal cell cancer and enhances tumor proliferation [9].

LINC01638 has been reported to be involved in the progression of several cancers, such as pancreatic ductal adenocarcinoma, non-small cell lung cancer, breast cancer and colorectal adenocarcinoma [10–13]. However, the role of LINC01638 in LSCC remains largely unknown. This study aimed to determine the functional mechanism of LINC01638 in LSCC. We found that LINC01638 was upregulated in LSCC tissues and contributed to LSCC progression through modulating miR-523-5p/BATF3 pathway. Our findings suggested that LINC01638 may be a novel therapeutic target for LSCC treatment.

## RESULTS

### Upregulated expression levels of LINC01638 in LSCC tissues

**Figure 1:**
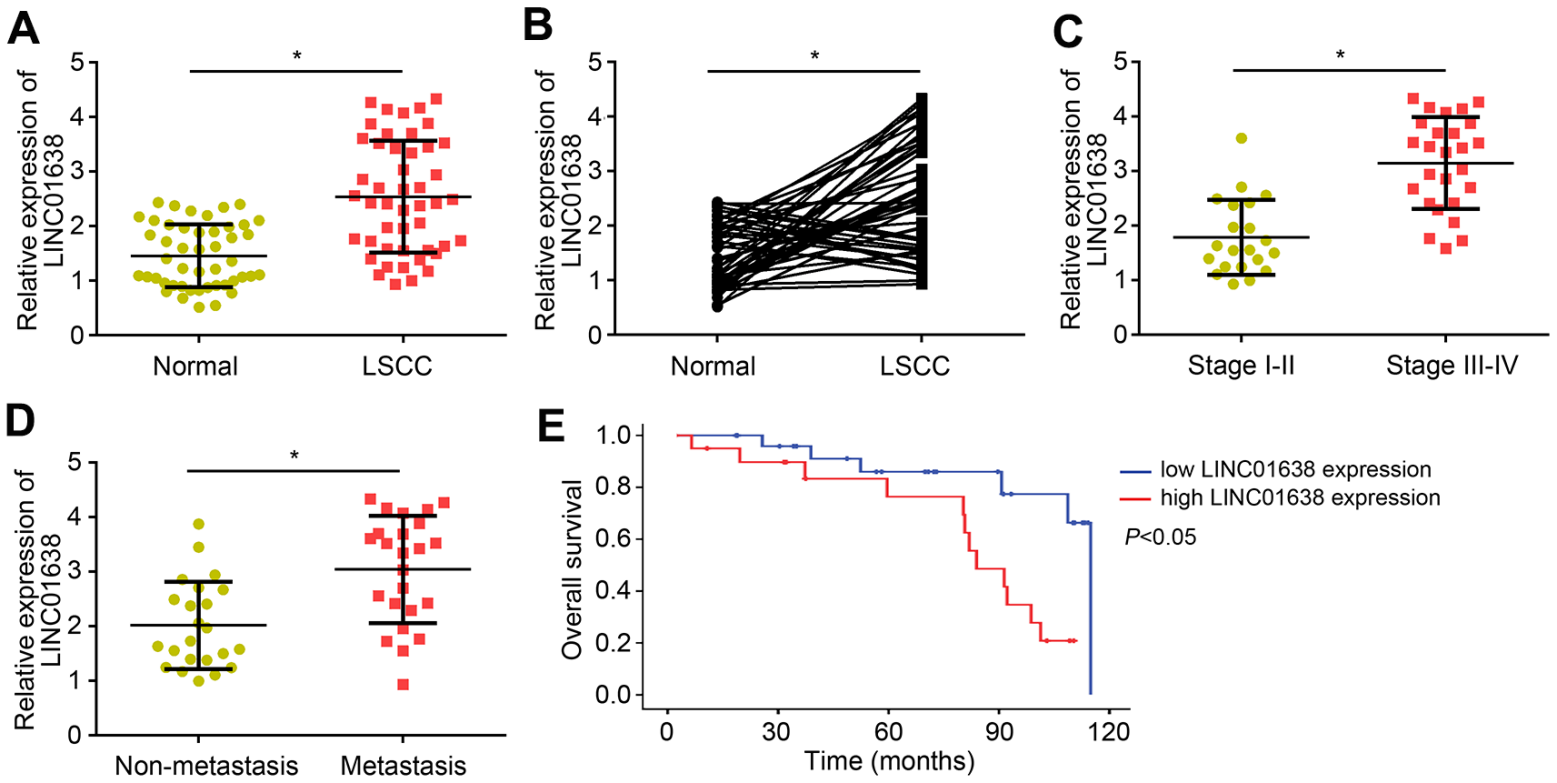
**Upregulated expression levels of LINC01638 in LSCC tissues.** (**A**) LINC01638 expression was upregulated in LSCC tissues compared to adjacent normal tissues. (**B**) LINC01638 was upregulated in most of the LSCC tissues compared to paired normal tissues. (**C**) LINC01638 was upregulated in advanced stages of LSCC tissues. (**D**) LINC01638 level was raised in LSCC tissues with lymph node metastasis. (**E**) LINC01638 overexpression was associated with a low survival rate. *P<0.05.

The expression of LINC01638 in LSCC was analyzed by qRT-PCR. Results indicated that LINC01638 level was raised in LSCC tissues compared to normal controls (Figure 1A). Notably, the expression of LINC01638 was upregulated in most of the LSCC tissues compared to their corresponding normal tissues (Figure 1B). Furthermore, LINC01638 expression was positively correlated with clinical stage and lymph node metastasis (Figure 1C, 1D). Then, survival rate was analyzed according to LINC01638 expression in LSCC tissues through the Kaplan-Meier method. Results showed that LINC01638 upregulation indicated a low survival rate (Figure 1E), suggesting that LINC01638 high expression predicted poor outcome.

### LINC01638 knockdown suppressed proliferation, migration and invasion

**Figure 2:**
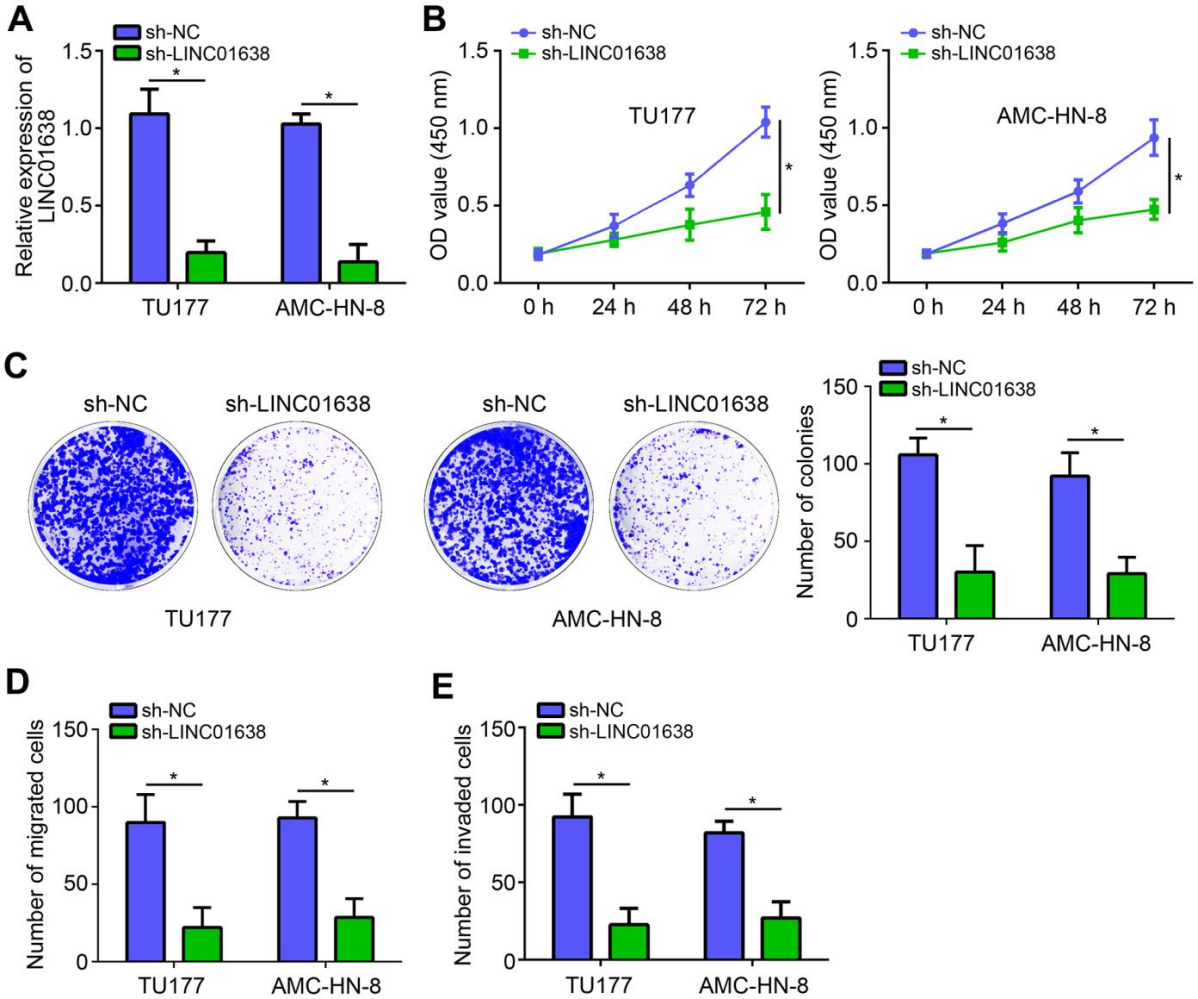
**LINC01638 knockdown suppressed proliferation, migration and invasion.** (**A**) LINC01638 shRNA decreased the expression of LINC01638 in TU177 and AMC-HN-8 cells. (**B**, **C**) Cell proliferation was evaluated by CCK8 and colony formation assays. (**D**, **E**) Transwell migration and invasion assays were performed to test migration and invasion. *P<0.05.

To analyze the function of LINC01638, two LSCC cell lines (TU177 and AMC-HN-8) were obtained and LINC01638 was knocked down by shRNA (Figure 2A). Using CCK8 assay, we found that LINC01638 knockdown significantly suppressed the proliferation of LSCC cells (Figure 2B). Colony formation assay further demonstrated that the colony number was decreased by LINC01638 silencing (Figure 2C). Additionally, transwell results showed that LINC01638 knockdown markedly reduced the cell migration and invasion (Figure 2D, 2E).

### LINC01638 was the sponge for miR-523-5p

**Figure 3:**
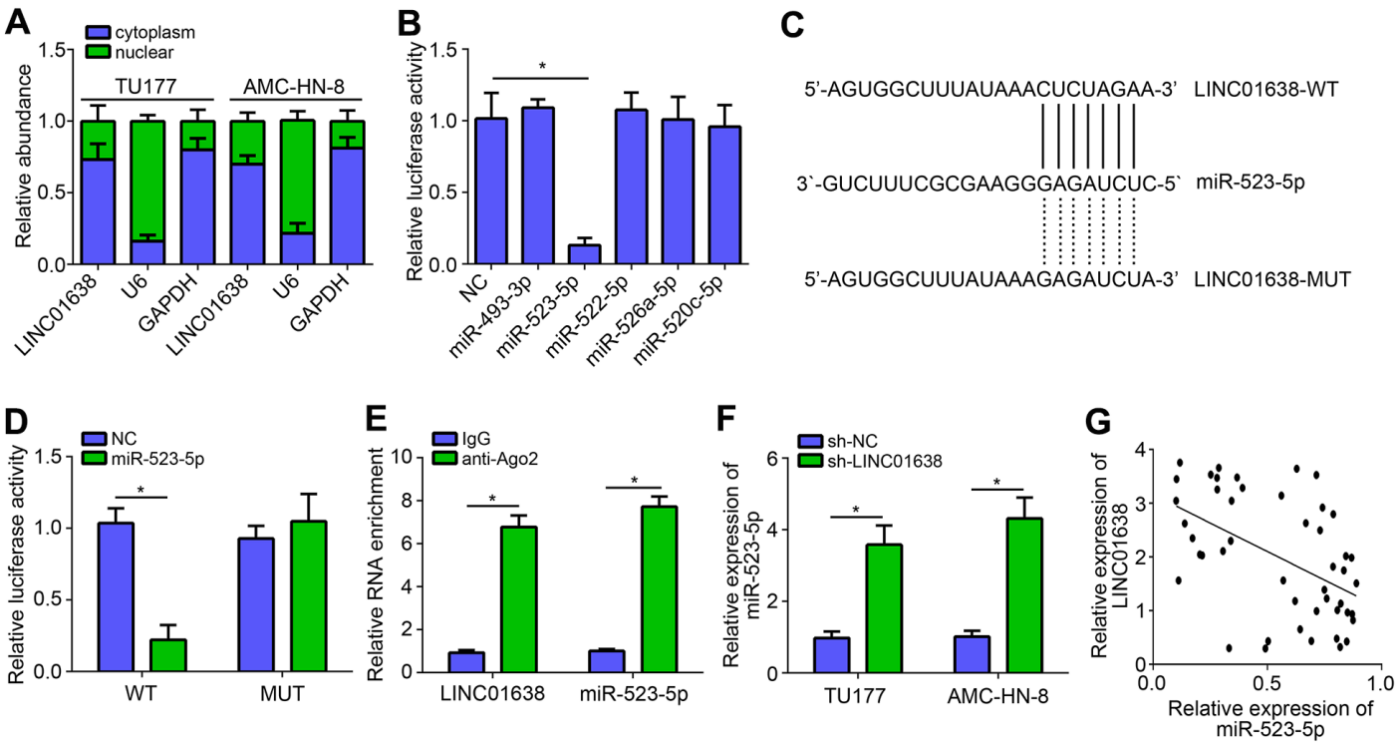
**LINC01638 was the sponge for miR-523-5p.** (**A**) Subcellular location of LINC01638 was analyzed by qRT-PCR. (**B**) Luciferase reporter assay identified that LINC01638 may be the sponge for miR-523-5p. (**C**) Predicted binding site and mutated binding site in LINC01638. (**D**) Luciferase reporter assay was performed. (**E**) RIP assay was conducted to examine the interaction between LINC01638 and miR-523-5p. (**F**) Relative expression of miR-523-5p was measured after LINC01638 knockdown. (**G**) Negative correlation was observed between LINC01638 and miR-523-5p expression in LSCC tissues. *P<0.05.

To investigate the molecular mechanism of LINC01638, we firstly analyzed its subcellular location by qRT-PCR. Results showed that LINC01638 was located in the cytoplasm (Figure 3A), indicating LINC01638 may be a miRNA sponge. Using bioinformatics analysis (miRDB online tool), five most potential candidates were identified. However, luciferase reporter assay showed that only miR-523-5p mimics inhibited the luciferase activity of LINC01638 (Figure 3B). Thus, we selected miR-523-5p for further validation. Mutant-LINC01638 (LINC01638-MUT) luciferase reporter vector was constructed (Figure 3C). Luciferase reporter assay validated the above conclusion again (Figure 3D). RIP assay further suggested that LINC01638 interacted with miR-523-5p (Figure 3E). Besides, we found that miR-523-5p expression was increased after LINC01638 knockdown (Figure 3F). Afterwards, it was observed that there was a negative correlation between LINC01638 and miR-523-5p expressions in LSCC tissues (Figure 3G).

### MiR-523-5p targeted BATF3 directly

**Figure 4:**
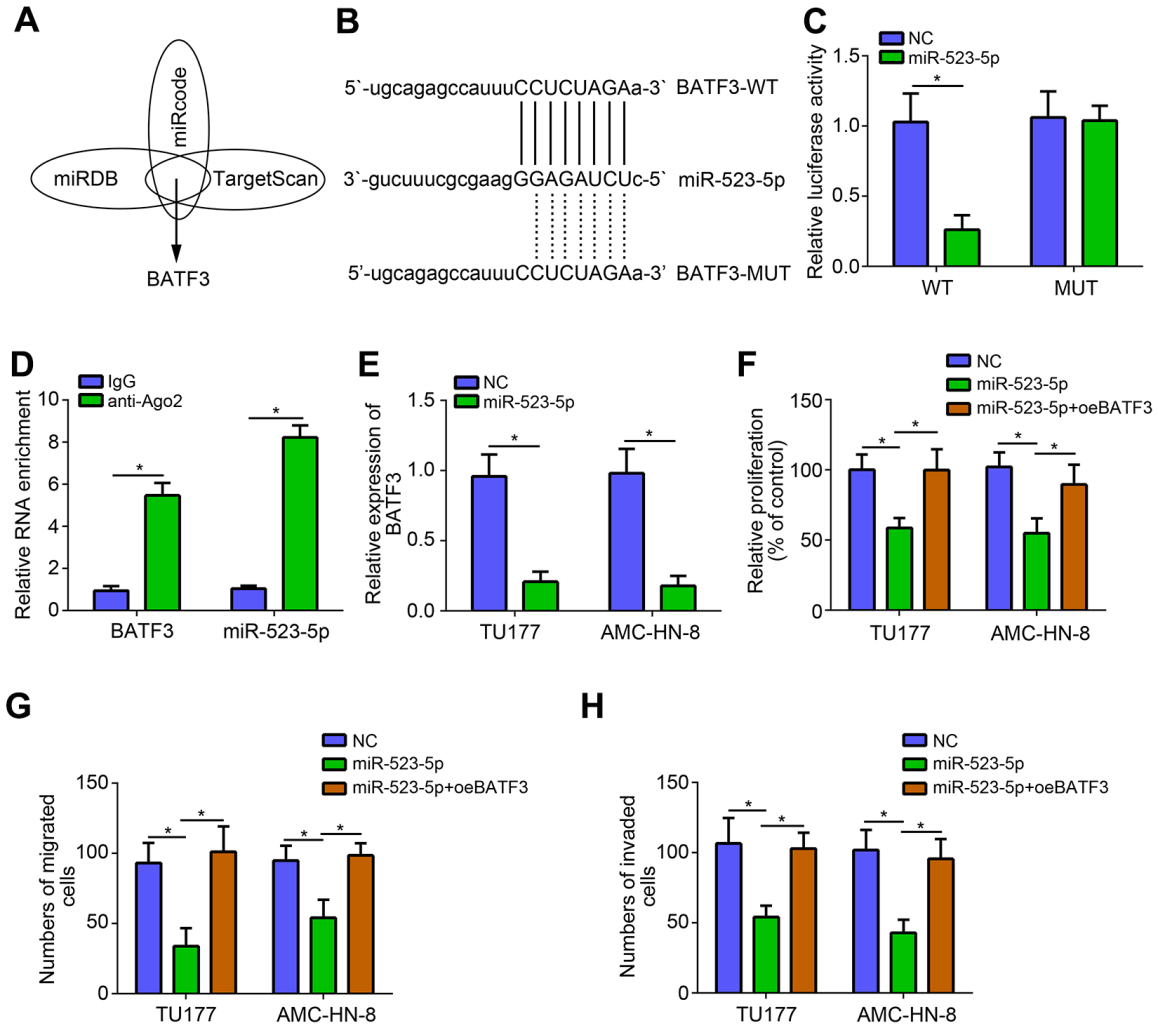
**miR-523-5p targeted BATF3 directly.** (**A**, **B**) Prediction of miR-523-5p targets by bioinformatics analysis. (**C**) Luciferase reporter assay was performed. (**D**) RIP assay was conducted to examine the interaction between miR-523-5p and BATF3. (**E**) miR-523-5p mimics suppressed the expression of BATF3. (**F**) CCK8 assay was utilized to test proliferation. (**G**, **H**) Transwell assay was performed to determine migration and invasion. *P<0.05.

Bioinformatics analysis was further conducted to predict the target of miR-523-5p (Figure 4A). BATF3 was identified as the most potential target. Similarly, luciferase reporter vectors were constructed (Figure 4B). Luciferase reporter assay showed that miR-523-5p mimics suppressed the activity of WT-BATF3 reporter (Figure 4C). RIP assay also suggested that miR-523-5p interacted with BATF3 (Figure 4D). Furthermore, qRT-PCR result showed that miR-523-5p mimics suppressed the expression of BATF3 (Figure 4E). To further explore the role of miR-523-5p, CCK8 and transwell assays were performed. Results showed that miR-523-5p mimics significantly repressed the proliferation, migration and invasion of LSCC cells (Figure 4F–4H). Notably, BATF3 overexpression eliminated the effects of miR-523-5p mimics (Figure 4F–4H).

### LINC01638 promoted LSCC progression through miR-523-5p/BATF3 pathway

**Figure 5:**
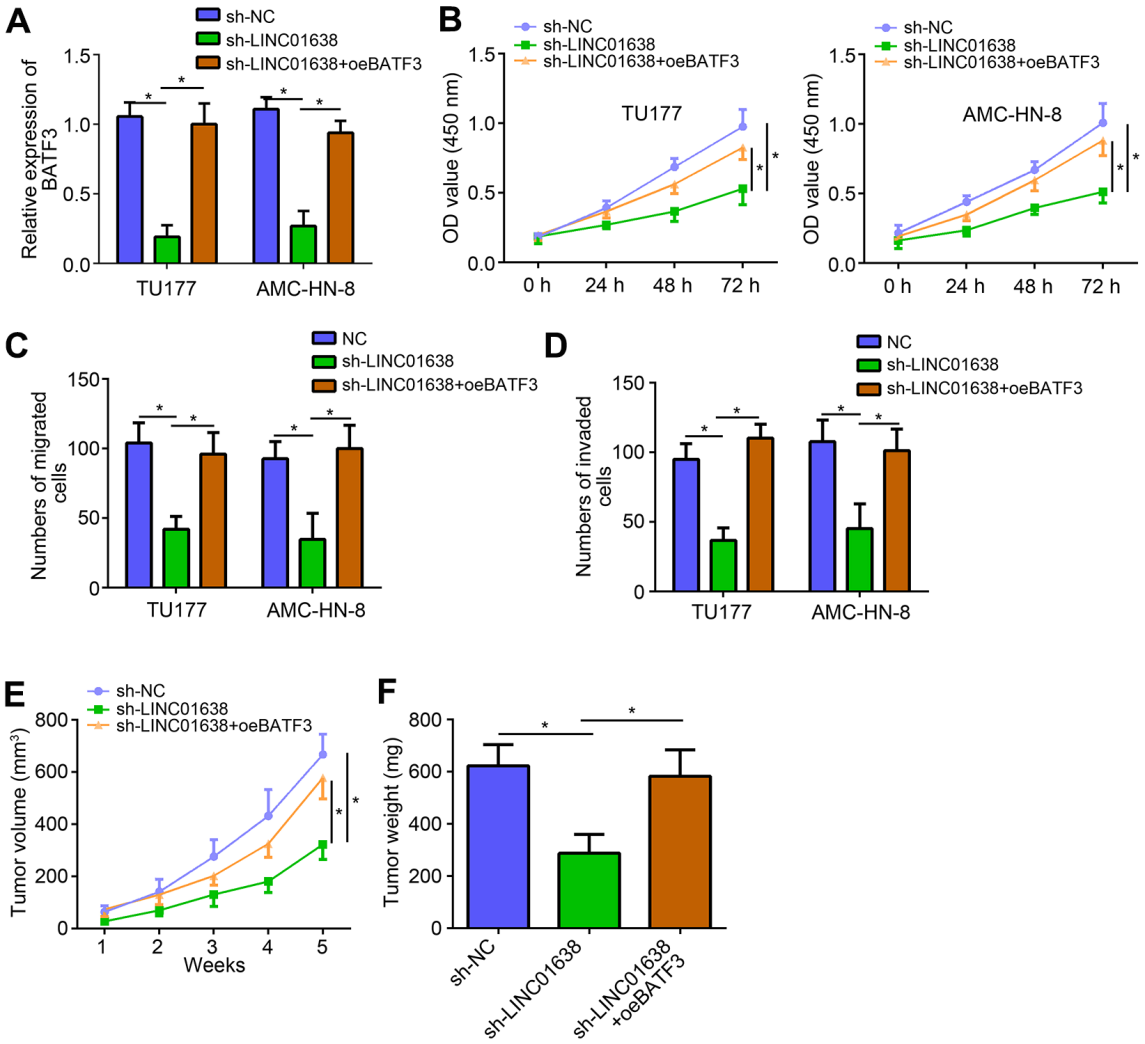
**LINC01638 promoted LSCC progression through miR-523-5p/BATF3 pathway.** (**A**) qRT-PCR analysis of BATF3 expression. (**B**) CCK8 assay was utilized to test proliferation. (**C**, **D**) Transwell assay was performed to determine migration and invasion. (**E**) Tumor volumes were measured every week. (**F**) After 5 weeks of injection, tumor weights were determined. *P<0.05.

We also noticed that LINC01638 knockdown suppressed BATF3 expression and miR-523-5p inhibitors reversed it (Figure 5A), suggesting that LINC01638 promoted BATF3 expression through inhibiting miR-523-5p.

Interestingly, we also found that overexpression of BATF3 reversed the inhibitory effects of LINC01638 knockdown on proliferation, migration and invasion (Figure 5B–5D). To further demonstrate the function of LINC01638/miR-523-5p/BATF3 axis, animal xenograft assay was performed. Result showed that LINC01638 knockdown suppressed the tumor volumes and weights *in vivo* (Figure 5E, 5F). Similarly, BATF3 overexpression also reversed the effects of LINC01638 knockdown *in vivo* (Figure 5E, 5F), indicating that LINC01638 promoted LSCC progression through miR-523-5p/BATF3 pathway.

## DISCUSSION

Increasing studies have supported the essential roles of lncRNAs in tumorigenesis, including LSCC. For example, lncRNA ST7-AS1 enhances proliferation, migration and invasion of LSCC through increasing the stability of CARM1 [14]. LncRNA PVT1 contributes to growth and invasion of LSCC through sponging miR-519d-3p [15]. LncRNA RGMB-AS1 facilitates the proliferation, migration and invasion in LSCC through modulating miR-22/NLRP3 pathway [16]. Therefore, it is important to make efforts to illustrate the molecular mechanism of lncRNA in LSCC progression, which will benefit the development of therapeutic targets.

LINC01638 was identified to be associated with human cancers as an oncogene. For instance, LINC01638 is firstly found to promote breast cancer progression through activating MTDH-Twist1 signaling [12]. Then, a study found that LINC01638 knockdown suppresses colorectal cancer cell growth [13]. Chen et al. reported that LINC01638 contributes to tumor growth of hepatocellular carcinoma [17]. Besides, Xiao et al. showed that LINC01638 is associated with melanoma recurrence [18]. Recently, some references indicated that LINC01638 positively regulates the development of prostate carcinoma, lung cancer and pancreatic ductal adenocarcinoma [10, 11, 19]. Nevertheless, its role in LSCC remains undetermined. In this work, we found that LINC01638 was highly expressed in LSCC tissues and correlated with clinical stage and metastasis. Moreover, LINC01638 high expression indicated poor prognosis. Furthermore, loss-of-function assay demonstrated that LINC01638 knockdown suppressed LSCC growth and metastasis *in vitro* and *in vivo*. Thus, LINC01638 acts oncogenic roles in LSCC progression.

Growing researches have demonstrated that LINC01638 interacts with miRNA to regulate tumorigenesis [6, 16]. For instance, lncRNA ZEB2-AS1 sponges miR-6840-4p to facilitate LSCC progression [20]. LncRNA NEAT1 enhances LSCC development by targeting miR-107/CDK6 axis [21]. Whether LINC01638 could be a sponge for miRNAs remains unclear. In our study, we found that LINC01638 was mainly expressed in the cytoplasm of LSCC cells. Through bioinformatics analysis, we identified several potential target miRNAs. We utilized luciferase reporter assay and RIP assay to demonstrate that LINC01638 interacted with miR-523-5p. Until now, miR-523-5p function has not been illustrated. Our results showed that miR-523-5p level was suppressed by LINC01638. And miR-523-5p mimics suppressed the proliferation, migration and invasion of LSCC cells, indicating that miR-523-5p is an anti-tumor miRNA.

The lncRNA-miRNA-mRNA regulatory axis has been widely acknowledged in cancer [21]. There is no report about the target of miR-523-5p. Through bioinformatics analysis, we identified that miR-523-5p may target BATF3. We then validated their binding through luciferase reporter assay and RIP assay. Besides, we found that BATF3 expression was inhibited by miR-523-5p. Moreover, BATF3 expression was decreased after LINC01638 silencing. And miR-523-5p inhibition rescued BATF3 expression. We demonstrated LINC01638 inhibited miR-523-5p to facilitate BATF3 expression. BATF3 is involved in several cancers, such as colorectal cancer, glioma and lymphoma [22–24]. The role of BATF3 in LSCC is elusive. In this research, we found that BATF3 overexpression restored the proliferation, migration and invasion of LINC01638-silenced LSCC cells *in vitro* and *in vivo*. Therefore, BATF3 promotes LSCC progression.

Conclusively, the present work illustrated that upregulation of LINC01638 promotes LSCC progression through modulating miR-523-5p/BATF3 axis, providing a novel insight onto the molecular mechanism underlying LSCC progression.

## MATERIALS AND METHODS

### Patients’ tissues

LSCC tissues and adjacent normal tissues were obtained from Shengjing Hospital of China Medical University. Informed consents were collected from every patient. Clinical data was listed in Table 1. This study was approved by the ethics committee of Shengjing Hospital of China Medical University. Tissues were stored in liquid nitrogen until use.

**Table 1:** Correlation between clinical data and LINC01638 expression.

**Feature**	**Low (n=26)**	**High (n=21)**	**P value**
Age (years)			0.774
<60	11	10	
≥60	15	11	
Gender			0.551
Male	14	14	
Female	12	7	
Stage			0.018
I-II	16	5	
III-IV	10	16	
Lymph node metastasis			0.003
No	18	5	
Yes	8	16	

### Cell culture and transfection

LSCC cell lines were purchased from American Type Culture Collection (ATCC; Manassas, VA, USA) and cultured using RPMI-1640 medium (Gibco), supplemented with 10% fetal bovine serum (Gibco). shRNA targeting LINC01638, miR-523-5p mimics, miR-523-5p inhibitors and negative controls were obtained from GenePharma (Shanghai, China). Transfection was performed using Lipofectamine 2000 (Invitrogen/Thermo Fisher Scientific, Inc., Waltham, MA, USA) according to the manufacturer’s instructions.

### qRT-PCR

RNA was extracted using Trizol reagent following the manufacturer’s instructions and transcribed into cDNA using the Transcriptor First Strand cDNA Synthesis kit (Roche, Basel, Switzerland) following the manufacturer’s protocol. Then qPCR was carried out using SYBR-Green Real-Time Master Mix (TaKaRa) on 7500 Real Time PCR System (Applied Biosystems, Foster City, CA, USA). Relative expression was normalized to GAPDH or U6 and calculated based on 2^−ΔΔCt^ method.

### Cell proliferation analysis

For CCK8 assay, cells were seeded into 96-well plates and cultured for indicated time points. Then 10 μL of CCK8 solution (Dojindo Laboratories, Kumamoto, Japan) was added and incubated for 2 h, followed by measurement of absorbance at 450 nm using a Microplate Reader (Bio-Rad, Hercules, CA, USA). For colony formation assay, cells were seeded into 6-well plates and cultured for 14 days. Then colonies were fixed with 70% ethanol and stained with 0.1% crystal violet. Numbers of colonies were finally counted.

### Transwell assay

For migration assay, cells were seeded into the non-Matrigel-coated upper chambers (8 μm pore; Corning Costar, Corning, NY, USA) with serum-free medium. The lower chamber was filled with FBS-containing medium. Cells were cultured for 24 h. Then migrated cells in the lower chamber was fixed with 70% ethanol and stained with 0.1% crystal violet. Cell numbers were finally counted using a light microscope. For invasion assay, cells were seeded into Matrigel-coated upper chambers and other steps were the same as migration assay.

### RNA immunoprecipitation (RIP) assay

The interactions among LINC01638, miR-523-5p and BATF3 were detected using RIP assay through the EZ-Magna RIP kit (Millipore, Billerica, MA, USA) following the manufacturer’s instructions.

### Luciferase reporter assay

The sequences of LINC01638 and BATF3 were inserted into pMIRGLO reporter vector (Promega). For luciferase reporter assay, the luciferase reporter and miR-523-5p mimics were transfected into TU177 cells and cultured for 48 h. Then the relative luciferase activity was measured using the Dual-Luciferase Reporter Assay System (Promega) according to the manufacturer’s protocol.

### Animal assay

5-week-old female nude (BALB/c-nude) mice from Charles River (Beijing, China) were randomly divided into three groups (5 mice per group). Then TU177 cells (5×10^6^) were subcutaneously injected into the right flanks of the mice. Tumor volumes were measured every week. After 5 weeks, tumor weights were examined. The animal experiment was approved by the ethics committee of Shengjing Hospital of China Medical University.

### Statistical analysis

The results were analyzed by GraphPad Prism version 6 and expressed as the means ± standard deviation. The differences between 2 groups were analyzed with the Student’s t-test. Differences between >2 groups were determined by one-way ANOVA followed by Tukey’s post hoc test. Pearson’s correlation analysis was utilized to determine expression correlation in tumor tissues. P<0.05 was considered to indicate a statistically significant difference.
